# A prospective appraisal of preoperative body mass index in D2-resected patients with non-metastatic gastric carcinoma and Siewert type II/III adenocarcinoma of esophagogastric junction: results from a large-scale cohort

**DOI:** 10.18632/oncotarget.19251

**Published:** 2017-07-12

**Authors:** Lei Huang, Zhi-Jian Wei, Tuan-Jie Li, Yu-Ming Jiang, A-Man Xu

**Affiliations:** ^1^ Department of General Surgery, The First Affiliated Hospital of Anhui Medical University, Hefei, China; ^2^ Department of General Surgery, Nanfang Hospital of Southern Medical University, Guangzhou, China; ^3^ Department of General Surgery, The Fourth Affiliated Hospital of Anhui Medical University, Hefei, China

**Keywords:** gastric cancer, adenocarcinoma of esophagogastric junction, body mass index, cancer-specific survival, prospective cohort study

## Abstract

**Objective:**

To prospectively investigate associations of presurgical body mass index (BMI) with clinicopathological factors and its prognostic significance in radically D2-resected patients with non-metastasized gastric cancer (GC) and Siewert type II/III adenocarcinoma of esophagogastric junction (AEG).

**Methods:**

A large prospective cohort consisting of radically-resected GC and AEG patients was analyzed. Follow-up was successful in 671 out of 700 patients, who were categorized into underweight (BMI<18.5), normal-weight (BMI=18.5-22.9), overweight (BMI=23-24.9), and obese (BMI≥25) groups according to Asian standards. BMI-associated factors were explored using multivariable logistic regression with adjustment. Cancer-specific survival analyses were conducted applying both univariable and multivariable Cox regression methods.

**Results:**

Pre-operation, higher hemoglobin levels and smaller anemia proportions were observed in larger BMI groups. Higher BMI tended to be associated with higher neutrophil-lymphocyte ratios (NLRs). Patients with higher BMI had smaller tumors and more often stage I tumors, but longer surgical time and postsurgical stay. In multivariable analyses, higher hemoglobin levels, upper tumor location, poorer differentiation, and higher NLR were significantly associated with higher BMI. Overall, survival analyses revealed no significant role of BMI. However, in further stratifications after adjustment, compared to patients with normal BMI, obese patients had better survival in women, but worse in those with AEG; underweight was associated with reduced mortality risk in tumors differentiated well to moderately; overweight patients had increased death hazard when having thrombocytopenia.

**Conclusion:**

Overall, preoperative BMI had limited prognostic significance in operated GC patients. However, under specific conditions (*e.g*., female, AEG, good differentiation, and thrombocytopenia), BMI might indicate postoperative survival.

## INTRODUCTION

Gastric cancer (GC) is one of the most common and lethal malignancies worldwide [1], especially in China [2]. Siewert type-II/III adenocarcinoma of esophagogastric junction (AEG), generally deemed as an independent cancer type, is nowadays becoming more prevalent in Asia [3]. Currently, D2 gastrectomy has been widely accepted as the standard surgical method in Asia, potentially benefiting survival together with advancement in adjuvant therapies [4].

Several clinicopathological factors have been revealed to be associated with GC and AEG prognosis besides patients’ demographic characteristics (*e.g*., age) [5, 6]. More advanced tumor stage [7], larger lymph node ratio [8], and poorer differentiation [9] might negatively predict survival. Proximal cancers are associated with a worse prognosis compared to distal ones [3, 10]. Tumor size is predictive of lymph node metastasis [11] and survival [12]. Increased preoperative neutrophil-lymphocyte ratio is positively associated with tumor progression and negatively with prognosis [13–16]. Hemoglobin level might impact treatment response rate and survival [17, 18]. Platelet count is associated with treatment response, but controversially with survival [16, 19].

With improvement of living standard, China is witnessing growing proportions of obese populations, associated with increasing rates of various chronic diseases and cancers including GC and AEG [20–22]. It is further indicated that BMI is associated with tumor location and differentiation [21–24]. Among the obese Chinese patients, fat usually gathers in abdomen, potentially increasing the difficulty of conducting abdominal surgery [25, 26]. Overweight and obesity might be associated with increased surgical time and positive-harvested lymph node ratio (LNR) which is negatively associated with survival [27]. However, some studies showed obesity did not significantly impact short-term perioperative outcomes [28–30]. Low BMI is associated with postoperative anemia in the long term [31]. Based on retrospective evidence, the association between BMI and postsurgical survival remains highly debatable [29, 32–34]. A small prospective study on mere GC patients only investigated the perioperative outcomes [35]. To the best of our knowledge, there are few prospective reports focusing on BMI in resected GC and AEG patients with a long-term follow-up, especially in the Chinese population. This study for the first time thoroughly investigated BMI-associated clinicopathological factors and its prognostic impact overall and in various subgroups in a large prospective Chinese cohort of GC and type-II/III AEG patients undergoing radical D2-gastrectomy.

## RESULTS

### Cohort characteristics

The overall and BMI group-specific patients’ clinicopathological data are shown in Table 1. Overall, a total of 671 resected patients were included in final analysis according to the eligibility criteria. The four BMI groups were comparable in gender, age, preoperative platelet, NLR, CEA and CA19-9, presurgical hospital stay, resection and digestive reconstruction types, and conduction of cholecystectomy and splenectomy. However, higher-BMI groups had higher levels of pretreatment hemoglobin (*P*=0.02) and smaller proportions of anemia (*P*=0.02). According to postoperative pathology, no significant differences were observed regarding tumor location, curvature, Borrman type in advanced cancers, pathological type, differentiation, early GC proportion, pN stage, neuro-invasion or thrombosis. However, significantly smaller tumors (*P*=0.01) and smaller proportions of large (>5 cm) tumors (*P*=0.02) were present in higher-BMI groups, where however trends towards smaller proportions of pT4a tumors and greater proportion of pT2 tumors were observed (*P*=0.06). Patients with higher BMI had also greater proportions of pTNM stage I tumors and overall smaller proportions of stage III cancers (*P*=0.03). Higher BMI was significantly associated with longer surgical time (*P*=0.00) and postoperative hospital stay (*P*=0.04), but the metastatic-harvested lymph node ratios were similar among the four groups.

**Table 1:** Clinicopathological data of the analyzed resected gastric cancer patients

Parameter	Value	*Overall*	*BMI group*				*χ^2^/F*	*P*
			*<18.5 kg/m^2^*	*18.5-23 kg/m^2^*	*23-25 kg/m^2^*	*≥25 kg/m^2^*		
n		671	84	357	118	112		
Gender	Male	515 (76.8)	62 (73.8)	281 (78.7)	85 (72.0)	87 (77.7)	0.01	0.918
Age (y)		62 ± 10	64 ± 12	62 ± 10	63 ± 9	63 ± 9	1.64	0.179
Age group	<60 ys	228 (34.0)	21 (25.0)	129 (36.1)	40 (33.9)	38 (33.9)	0.99	0.321
	60-69 ys	281 (41.9)	37 (44.0)	145 (40.6)	50 (42.4)	49 (43.8)		
	≥70 ys	162 (24.1)	26 (31.0	83 (23.3)	28 (23.7)	25 (22.3)		
Weight (kg)		59 ± 9	48 ± 5	56 ± 6	63 ± 6	72 ± 8	300.25	**0.000**
Height (cm)		164 ± 7	167 ± 7	164 ± 7	163 ± 8	162 ± 8	7.22	**0.000**
BMI (kg/m^2^)		22.0 ± 3.2	17.4 ± 9.5	20.8 ± 1.2	24.0 ± 0.5	27.3 ± 2.1	-	-
BMI group	<18.5 kg/m^2^	84 (12.5)	84 (100.0)	-	-	-	-	-
	18.5-22.9 kg/m^2^	357 (53.2)	-	357 (100.0)	-	-		
	23-24.9 kg/m^2^	118 (17.6)	-	-	118 (100.0)	-		
	≥25 kg/m^2^	112 (16.7)	-	-	-	112 (100.0)		
Presurgical hemoglobin (g/L)		116 ± 27	112 ± 25	114 ± 27	120 ± 26	121 ± 27	3.25	**0.021**
Presurgical anemia	Yes	258 (41.9)	32 (44.4)	152 (46.2)	40 (35.4)	34 (33.3)	5.866	**0.016**
Presurgical neutrophil-lymphocyte ratio	2.59 ± 2.72	2.30 ± 0.95	2.57 ± 1.82	2.32 ± 1.48	3.17 ± 5.54	2.20	0.087	
Presurgical platelet (×10^9^/L)		199 ± 79	200 ± 92	204 ± 82	191 ± 72	192 ± 62	1.11	0.344
Presurgical thrombocytopenia	Yes	126 (20.5)	21 (29.2)	60 (18.2)	25 (22.1)	20 (19.8)	0.40	0.525
Presurgical CEA (μg/L)		7.9 ± 20.0	8.0 ± 13.2	9.0 ± 22.3	4.5 ± 6.1	7.9 ± 25.0	0.88	0.450
Presurgical CA19-9 (kU/L)		44 ± 127	81 ± 217	36 ± 93	54 ± 160	30 ± 92	1.97	0.118
Presurgical hospital stay (d)		6 ± 4	7 ± 5	6 ± 4	6 ± 4	6 ± 4	0.44	0.722
Surgery type	Open	650 (96.9)	82 (97.6)	350 (98.0)	110 (93.2)	108 (96.4)	2.10	0.147
Resection type	Distal gastrectomy	153 (22.8)	21 (25.0)	80 (22.4)	27 (22.9)	25 (22.3)	0.19	0.909
	Total gastrectomy	479 (71.4)	60 (71.4)	253 (70.9)	87 (73.7)	79 (70.5)		
	Proximal gastrectomy	39 (5.8)	3 (3.6)	24 (6.7)	4 (3.4)	8 (7.1)		
Reconstruction type	Roux-en-Y	574 (85.5)	70 (83.3)	306 (85.7)	105 (89.0)	93 (83.0)	2.12	5.49
	Bilroth-I	51 (7.6)	7 (8.3)	27 (7.6)	7 (5.9)	10 (8.9)		
	Bilroth-II	28 (4.2)	6 (7.2)	14 (3.9)	4 (3.4)	4 (3.6)		
	Esophagogastrostomy	18 (2.7)	1 (1.2)	10 (2.8)	2 (1.7)	5 (4.5)		
Cholecystectomy	Yes	42 (6.3)	3 (3.6)	24 (6.7)	7 (5.9)	8 (7.1)	0.46	0.499
Splenectomy	Yes	17 (2.5)	3 (3.6)	11 (3.1)	2 (1.7)	1 (0.9)	2.25	0.134
Tumor location in stomach	EGJ	310 (46.2)	29 (34.5)	166 (46.5)	64 (54.2)	51 (45.5)	2.62	0.105
	Cardia & fundus	39 (5.8)	5 (6.0)	21 (5.9)	5 (4.2)	8 (7.1)		
	Fundus	21 (3.1)	2 (2.4)	16 (4.5)	0 (0.0)	3 (2.7)		
	Fundus & body	9 (1.3)	3 (3.6)	4 (1.1)	1 (0.9)	1 (0.9)		
	Body	71 (10.6)	13 (15.5)	28 (7.8)	12 (10.2)	18 (16.1)		
	Body & antrum	15 (2.2)	1 (1.2)	11 (3.1)	3 (2.5)	0 (0.0)		
	Antrum & pylorus	176 (26.2)	26 (31.0)	92 (25.8)	30 (25.4)	28 (25.0)		
	Full stomach	30 (4.5)	5 (6.0)	19 (5.3)	3 (2.5)	3 (2.7)		
Curvature	Small	633 (94.3)	77 (91.7)	337 (94.4)	111 (94.1)	108 (96.4)	1.48	0.223
Borrmann type	I	24 (4.2)	3 (4.2)	16 (5.2)	0 (0.0)	5 (5.4)	1.53	0.216
	II	360 (63.3)	41 (57.8)	189 (61.0)	69 (72.6)	61 (65.6)		
	III	160 (28.1)	22 (31.0)	92 (29.7)	23 (24.2)	23 (24.7)		
	IV	25 (4.4)	5 (7.0)	13 (4.2)	3 (3.2)	4 (4.3)		
Pathological type	Adenocarcinoma	607 (91.3)	76 (90.5)	325 (91.8)	107 (91.5)	99 (90.0)	0.13	0.988
	Signetring cell carcinoma	11 (1.7)	2 (2.4)	6 (1.7)	0 (0.0)	3 (2.7)		
	Squamous cell carcinoma	5 (0.8)	1 (1.2)	2 (0.6)	1 (0.9)	1 (0.9)		
	Mucinous cell carcinoma	42 (6.3)	5 (6.0)	21 (5.9)	9 (7.7)	7 (6.4)		
Tumor length (cm)		5.1 ± 2.9	5.5 ± 3.1	5.3 ± 3.0	4.5 ± 2.2	4.7 ± 2.8	3.95	**0.008**
Tumor length >5 cm	Yes	253 (38.2)	37 (44.6)	144 (40.9)	36 (31.0)	36 (32.1)	5.66	**0.017**
Tumor stage	Early	92 (14.0)	11 (13.1)	45 (12.6)	20 (17.0)	16 (14.3)	0.49	0.486
pT	1	92 (14.0)	11 (13.4)	45 (12.8)	20 (17.4)	16 (14.8)	3.69	0.055
	2	59 (9.0)	5 (6.1)	26 (7.4)	13 (11.3)	15 (13.9)		
	3	34 (5.2)	7 (8.5)	15 (4.3)	2 (1.7)	10 (9.3)		
	4a	412 (62.7)	54 (65.9)	231 (65.6)	66 (57.4)	61 (56.5)		
	4b	60 (9.1)	5 (6.1)	35 (9.9)	14 (12.2)	6 (5.6)		
pN	0	267 (40.3)	27 (32.9)	140 (40.0)	50 (43.5)	50 (45.1)	2.20	0.138
	1	147 (22.2)	21 (25.6)	84 (23.7)	23 (20.0)	19 (17.1)		
	2	149 (22.5)	18 (22.0)	77 (21.8)	25 (21.7)	29 (26.1)		
	3a	87 (13.2)	13 (15.9)	46 (13.0)	16 (13.9)	12 (10.8)		
	3b	12 (1.8)	3 (3.7)	7 (2.0)	1 (0.9)	1 (0.9)		
pTNM stage	IA	82 (13.0)	7 (8.6)	41 (12.2)	18 (16.5)	16 (15.1)	4.98	**0.026**
	IB	42 (6.6)	4 (4.9)	18 (5.3)	10 (9.2)	10 (9.4)		
	IIA	31 (4.9)	7 (8.6)	14 (4.2)	5 (4.6)	5 (4.7)		
	IIB	138 (21.8)	17 (21.0)	77 (22.9)	21 (19.3)	23 (21.7)		
	IIIA	107 (16.9)	13 (16.1)	63 (18.7)	15 (13.8)	16 (15.1)		
	IIIB	113 (17.9)	13 (16.1)	58 (17.2)	19 (17.4)	23 (21.7)		
	IIIC	120 (19.0)	20 (24.7)	66 (19.6)	21 (19.3)	13 (12.2)		
Differentiation grade	Well	20 (3.3)	1 (1.3)	10 (3.1)	2 (1.9)	7 (6.9)	0.35	0.552
	Well-moderate	10 (1.6)	3 (4.0)	4 (1.2)	3 (2.9)	0 (0.0)		
	Moderate	194 (31.9)	21 (28.0)	115 (35.2)	33 (31.4)	25 (24.8)		
	Moderate-poor	153 (25.2)	20 (26.7)	80 (24.5)	33 (31.4)	20 (19.8)		
	Poor	217 (35.7)	29 (38.7)	110 (33.6)	33 (31.4)	45 (44.6)		
	Undifferentiated	14 (2.3)	1 (1.3)	8 (2.5)	1 (1.0)	4 (4.0)		
Neuro-invasion	Yes	13 (1.9)	2 (2.4)	7 (2.0)	2 (1.7)	2 (1.8)	0.10	0.758
Tumor thrombosis	Yes	49 (7.3)	5 (6.0)	31 (8.7)	8 (6.8)	5 (4.5)	0.91	0.339
Surgical duration (min)		175 ± 55	164 ± 48	168 ± 49	184 ± 54	197 ± 69	9.25	**0.000**
Positive-harvested lymph node ratio	0.25 ± 0.31	0.27 ± 0.29	0.25 ± 0.32	0.25 ± 0.30	0.25 ± 0.31	0.14	0.938	
Postsurgical hospital stay (d)		12 ± 7	12 ± 5	12 ± 7	12 ± 4	14 ± 8	2.88	**0.036**
Follow-up^1^ (mo)		71 (69-74)	70 (64-74)	71 (67-74)	71 (70-75)	74 (70-76)	-	-

Enumeration data are shown as n (percentage [%]), and measurement data as mean ± standard deviation.

^1^Calculated using the reverse Kaplan-Meier method and shown as median (interquartile).

BMI, body mass index; CEA, carcino-embryonic antigen; CA19-9, cancer antigen 19-9; EGJ, esophagogastric junction.

### Association of BMI with clinicopathological parameters

The association of BMI with preoperative demographical and clinical characteristics using multivariable logistic regression are shown in Table 2. Greater BMI was significantly associated with higher presurgical hemoglobin levels (*P*=0.02), more proximal tumor locations (*P*=0.01), poorer differentiation grades (*P*=0.02), and higher NLR (*P*=0.02). However, gender, age, platelet count, tumor curvature, pathology, length, pT stage, metastatic-harvested lymph node ratio, neuro-invasion, and tumor thrombosis were not significantly associated with preoperative BMI.

**Table 2:** Association of body mass index with presurgical clinicopathological factors in resected gastric cancer patients using multivariable logistic regression

Parameter	Odds ratio	95% confidence interval	Wald *χ^2^*	*P*
Gender	1.02	0.68-1.53	0.01	0.926
Age	1.02	1.00-1.03	2.56	0.110
Presurgical hemoglobin	1.01	1.00-1.02	5.26	**0.022**
Presurgical platelet	1.00	1.00-1.00	0.02	0.899
Tumor location	0.91	0.86-0.97	7.57	**0.006**
Curvature	0.72	0.34-1.54	0.70	0.401
Pathology	0.75	0.41-1.37	0.90	0.343
Tumor length	0.79	0.53-1.17	1.39	0.239
pT	0.88	0.75-1.03	2.43	0.119
Positive-harvested lymph node ratio	1.38	0.79-2.41	1.25	0.264
Differentiation grade	1.23	1.03-1.45	5.37	**0.021**
Neuro-invasion	0.84	0.26-2.70	0.08	0.775
Tumor thrombosis	0.89	0.46-1.73	0.11	0.738
Neutrophil-lymphocyte ratio	1.08	1.01-1.16	5.30	**0.021**

Odds ratios (ORs) indicating associations of sequential body mass index groups (<18.5 kg/m^2^, 18.5-23 kg/m^2^, 23-25 kg/m^2^, and ≥25 kg/m^2^) with clinicopathological characteristics for resected gastric cancer patients are shown as point estimate (95% confidence interval). ORs were calculated using the multivariable logistic regression model adjusting for the factors listed in the left-most column.

### CSS-associated factors

The associations of CSS with clinicopathological factors are shown in Table 3. The median follow-up was 71 (interquartile, 69-74) months. Using univariable Cox regression analysis, older ages (*P*=0.00), higher NLRs (*P*=0.00), resection (*P*=0.02) and reconstruction types (*P*=0.03), splenectomy (*P*=0.03), larger tumor size (*P*=0.00), more advanced pT stage (*P*=0.00), larger positive-harvested lymph node ratio (*P*=0.00), poorer differentiation grades (*P*=0.00), neuro-invasion (*P*=0.02), and tumor thrombosis (*P*=0.00) were significantly associated with poorer survivals. Applying multivariable Cox regression models, older age (*P*=0.00), tumor location (*P*=0.02), larger tumor size (*P*=0.02), higher pT stage (*P*=0.00), and larger metastatic-harvested lymph node ratio (*P*=0.00) were significant independent postoperative CSS-indicators. However, BMI did not show any prognostic significances overall either in univariable (*P*=0.28) or multivariable (*P*=0.30) analysis.

**Table 3:** Association of cancer-specific survival with clinicopathological factors in resected gastric cancer patients using univariable and multivariable Cox regression models

Parameter	Univariate analysis				Multivariate analysis			
	Hazard ratio	95% confidenceinterval	*χ^2^*	*P*	Hazard ratio	95% confidenceinterval	*χ^2^*	*P*
Gender	0.94	0.73-1.21	0.24	0.623	1.07	0.80-1.44	0.19	0.661
Age	1.02	1.01-1.03	12.36	**0.000**	1.03	1.02-1.04	16.34	**0.000**
**Body mass index**	0.98	0.95-1.02	1.16	0.281	1.02	0.98-1.06	1.08	0.300
Presurgical hemoglobin	1.00	0.99-1.00	1.72	0.190	1.00	1.00-1.01	1.55	0.213
Presurgical platelet	1.00	1.00-1.00	1.30	2.55	1.00	1.00-1.00	0.22	0.641
Neutrophil-lymphocyte ratio	1.04	1.02-1.07	10.59	**0.001**	1.02	0.99-1.06	1.66	0.198
Surgery type	0.83	0.43-1.61	0.30	0.586	0.55	0.17-1.74	1.04	0.307
Resection type	1.26	1.03-1.54	5.26	**0.022**	1.36	0.98-1.89	3.30	0.069
Reconstruction type	0.81	0.67-0.98	4.78	**0.029**	1.09	0.87-1.37	0.52	0.473
Cholecystectomy	0.84	0.53-1.33	0.55	0.457	1.02	0.63-1.65	0.00	0.945
Splenectomy	1.85	1.06-3.22	4.72	**0.030**	1.31	0.71-2.44	0.73	0.392
Tumor position	0.99	0.96-1.03	0.09	0.769	1.07	1.01-1.13	5.70	**0.017**
Curvature	0.85	0.52-1.38	0.45	0.501	0.93	0.51-1.69	0.06	0.806
Pathological type	1.03	0.93-1.14	0.30	0.584	0.82	0.36-1.89	0.22	0.641
Tumor length	1.17	1.13-1.20	94.56	**0.000**	1.07	1.01-1.12	5.81	**0.016**
pT	1.65	1.47-1.85	69.95	**0.000**	1.39	1.20-1.61	18.49	**0.000**
Positive-harvested lymph node ratio	3.88	3.16-4.77	168.05	**0.000**	2.88	2.11-3.93	44.05	**0.000**
Differentiation grade	1.20	1.08-1.34	11.80	**0.001**	1.07	0.94-1.23	0.07	0.959
Neuro-invasion	2.01	1.10-3.67	5.16	**0.023**	1.12	0.56-2.25	0.10	0.750
Tumor thrombosis	1.94	1.39-2.72	14.85	**0.000**	1.11	0.73-1.68	0.22	0.640

Continuous data were applied where applicable.

### Association of BMI with CSS in various subgroups

The association of BMI with postoperative CSS in different stratifications using adjusted multivariable Cox proportional hazard regressions are shown in Table 4. With the normal BMI group of 18.5-23 kg/m^2^ as the standard, obesity significantly reduced death risk in female (hazard ratio [HR]=0.38; 95% confidence interval [CI]=0.19-0.97), but increased risk among patients with AEG (HR=1.53, 95% CI=1.01-2.39). Overweight significantly increased mortality risk in patients with presurgical thrombocytopenia (HR=3.03, 95% CI=1.12-8.17). Underweight significantly reduced death risk among people with well, well-moderately, and moderately differentiated tumors. All the other association findings were statistically insignificant.

**Table 4:** Association of body mass index with cancer-specific survival in resected gastric cancer patients using multivariable Cox regression

Parameter	Value	Body mass index (kg/m^2^), HR (95% CI)		
		*<18.5 vs. 18.5-23*	*23-25 vs. 18.5-23*	*≥25 vs. 18.5-23*
Comprehensive		0.85 (0.57-1.27)	1.11 (0.79-1.55)	1.13 (0.80-1.60)
Gender	Male	0.94 (0.61-1.45)	1.25 (0.85-1.82)	1.24 (0.86-1.21)
	Female	0.49 (0.20-1.20)	0.74 (0.33-1.66)	**0.38** (0.19-0.97)
Age group	<60 ys	0.88 (0.36-2.16)	0.93 (0.48-1.79)	0.81 (0.35-1.87)
	60-69 ys	0.65 (0.34-1.23)	1.00 (0.56-1.79)	1.30 (0.78-2.14)
	≥70 ys	0.91 (0.43-1.91)	0.84 (0.41-1.72)	1.01 (0.49-2.09)
Tumor position	Esophagogastric junction	0.82 (0.45-1.21)	1.11 (0.72-1.71)	**1.53** (1.01-2.39)
	Non-esophagogastric junction	0.91 (0.48-1.72)	0.85 (0.46-1.58)	0.63 (0.32-1.24)
Differentiation grade	Well, well-moderate & moderate	**0.37** (0.15-0.89)	1.73 (0.93-3.20)	1.41 (0.72-2.76)
	Moderate-poor, poor & undifferentiated	0.94 (0.60-1.47)	0.90 (0.61-1.34)	1.06 (0.71-1.59)
pTNM stage	I-II	0.79 (0.33-1.91)	0.79 (0.40-1.57)	0.83 (0.42-1.67)
	III	0.78 (0.48-1.25)	1.14 (0.73-1.76)	1.31 (0.85-2.02)
Presurgical anemia	No	0.70 (0.38-1.29)	1.07 (0.66-1.73)	1.27 (0.78-2.08)
	Yes	0.89 (0.48-1.65)	1.02 (0.60-1.72)	0.88 (0.49-1.58)
Presurgical thrombocytopenia	No	0.73 (0.46-1.16)	1.03 (0.70-1.52)	1.17 (0.80-1.72)
	Yes	1.55 (0.46-5.25)	**3.03** (1.12-8.17)	1.60 (0.43-5.47)

Hazard ratios (HRs) indicating association between body mass index and gastric cancer-specific survival are presented as point estimate (95% confidence interval) after adjustment for gender, age group, surgery type, gastrectomy type, digestive reconstruction type, cholecystectomy, splenectomy, hepatectomy, tumor location, curvature, pathology, length, pT, pN, differentiation, neuro-invasion, thrombosis, anemia and thrombocytopenia, overall and in each stratification by clinicopathological parameters of the patients. HRs were calculated using the multiple Cox regression model with adjustment, and are statistically significant when shown in bold.

HR, hazard ratio; CI, confidence interval.

## DISCUSSION

Nowadays with the continuous improvement in living standard, the proportions of overweight and obese people keep increasing dramatically throughout the world especially in the Asia-Pacific region, making it a major health problem [36]. Higher BMI could lead to many chronic diseases including hypertension, diabetes and hyperlipidemia, and various malignancies like pancreatic cancer, colorectal cancer, breast cancer, liver cancer, lung cancer, prostate cancer, thyroid cancer, ovarian cancer, cervical cancer and leukemia, where it might also be prognostically significant [22, 32, 36–40]. Meanwhile, under rising socioeconomical pressure, still a significant number of people are underweight. Abnormal weight is closely associated with GC genesis, and might as well impact prognosis [24, 41, 42]. Up till now, little prospective evidence has been reported concerning BMI in resected upper digestive malignancies. Especially, whether underweight, which usually indicates disease progression and advanced stage [43], is prognostically significant remains scarcely explored. This study prospectively investigated BMI in resected GC and type II/III AEG using a large Chinese cohort with long follow-up periods. Several interesting BMI-associated clinicopathological factors were revealed. Although overall, BMI did not play a significant prognostic role, it was associated with CSS in several subgroups.

In this study, larger preoperative BMI associated with higher hemoglobin levels, lower anemia proportions, larger NLR, more proximal tumor locations, poorer tumor differentiation and more advanced pTNM stage, but interestingly with smaller tumor size. BMI reflects overall nutrition and immunity statuses [44]. Overweight and obese populations are less likely anemic [45] which might be associated with the indicated better nutritional status, and the positive association between BMI and hemoglobin levels were stronger in men [46]. Anemia is a negative prognostic marker in GC [47, 48]. Obesity is associated with chronic low-grade inflammation and immunological disorders [38, 49, 50], which correlates with tumor progression. High NLR is a risk factor for GC, and positively associates with tumor size and stage [51]. It is also negatively prognostic in GC [14, 52, 53] and AEG [54, 55]. Notably, in this study tumor pTNM stage and size but not differentiation grade were differently distributed in the four BMI groups using *χ^2^* test. However, when applying BMI as a continuous variable and using the multivariable logistic regression models, stage and size were not associated with BMI, but an association of tumor differentiation was observed. These findings however require further validation or clarification. More advanced tumor stage [7], larger lymph node ratio [8], larger tumor size [12], and poorer differentiation [9] might also negatively predict survival. A small-scale retrospective study also supported the positive association of BMI with tumor stage, but not with tumor location [56]. The higher BMI-associated poorer differentiation observed might be explained by the disrupted metabolic status of malignant cells, making them more aggressive in biological behavior. Findings in other cancer entities are controversial but interesting. In breast cancer, it was also found that obesity at the time of diagnosis was associated with more advanced tumor stages and poorly differentiated grade [57, 58], which was however not supported by other investigations [59]. Higher BMI was associated with non-organ-confined prostate cancers [60]. While in penile cancer, no association between BMI and cancer stage was observed [61]. In esophageal carcinoma, patients with high BMI tend to have lower stage at diagnosis [62]. AEG might be detected at an earlier stage than non-AEG due to early local obstructive symptoms, partly explaining the higher BMI. However, tumors located in gastric antrum/pylorus might be more insidious, and could grow to a relatively large mass causing obstruction, further leading to malnutrition. Notably, higher BMI is a well-established risk factor especially for proximal GC and AEG compared to distal GC [3, 23, 24, 42, 63], while the underlying mechanisms warrant further investigation. Type II/III AEG might associate with poorer survival compared to non-AEG GC, after adjustment of survival-associated covariates [3]. Our results further supported that age, NLR, resection and reconstruction types, splenectomy, tumor length, pT stage, positive-harvested lymph node ratio, differentiation, neuro-invasion and tumor thrombosis were associated with CSS, and that age, tumor position, size, pT stage, and lymph node ratio were independent prognostic markers. Taking all these into consideration, the prognostic significance of BMI in GC and AEG might be complicated and clinicopathological parameter-dependent.

Overall, neither univariable nor multivariable analysis revealed any significant association between BMI and CSS in this investigation. However, in further subgroup analyses, interestingly, overweight/obesity increased HR in patients with type II/III AEG and those with presurgical thrombocytopenia, but decreased risk in female. Underweight decreased mortality risk in well- to moderately-differentiated cancers. No age group-, pTNM stage-, and anemia-specific CSS differences were detected in relation to BMI. Previous retrospective evidence concerning the prognostic role of BMI in GC remains controvertial. Some supported that higher pretreatment BMI did not meaningfully predict postoperative survival [30, 32, 44, 56, 64–66], some indicated a positive association between BMI and postoperative survival [33, 34, 67], while others suggested a negative correlation [68]. The different findings could partly due to the fact that some other researches did not adjust confounding factors as thoroughly as we did, which could then hopefully reveal the true associations. Several studies consistently showed underweight was negatively prognosis-indicative [29, 43, 69]. Interestingly, obesity is only associated with increased risk of AEG but not non-cardia GC [23, 24, 42, 63], which is consistent with the subsite-specific findings here that obesity only increased the death risk in AEG. The observed obesity-associated increased risk of mortality could be possibly explained by the more strongly and earlier disrupted metabolic and immune status in the population. Effect of obesity on GC might be gender-specific: it is associated with GC in men, where it was associated with increased incidence of early and well- to moderately-differentiated GC, while in women it was associated with gastric dysplasia [41]. The observation that obesity seemed protective in female GC patients is noteworthy. The premenopausal female sex is a known protective factor against various malignancies, potentially due to the effect of the sex hormones. In obese females, the endocrinal and metabolic statuses are disorganized, which however might up-regulate the protective hormone levels or facilitate the underlying functions. These however need to be validated in further investigations. Platelets are associated with inflammation and tumor progression in GC [70, 71], and the combination with higher BMI might indicate greater tumor invasiveness. In less invasive tumors with good to moderate differentiation, underweight appears protective, potentially indicating the theory ‘starve-tumor-to-death’ works better in less aggressive cancers [72]. Besides, survival patterns in patients with tumors of more benign differentiation might be more in line with the normal population, where underweight could be beneficial to some extent. The underlying mechanisms through which BMI might prognostically significant are worth further clarification.

In this research, patients with higher BMI especially those obese had significantly longer surgical time and postoperative hospital stay, while the metastatic-harvested lymph node ratios were similar in the four BMI groups. Due to potentially different inclusion criteria and regions, researches revealed controversial association of overweight/obesity and surgical parameters including operation duration and lymph node ratio [28–30, 65, 73]. Overweight and obesity might be associated with more comorbidities [74], and potentially increase the complexity and difficulty of gastric surgery [32, 64, 68]. Both underweight and overweight increased postoperative complications [68, 75]. Notably, visceral obesity condition and body-shape index (BSI) might also well predict short-term post-gastrectomy outcomes [76, 77]. The impact of BMI on surgical outcomes might decrease with advancement in surgical skills and techniques, and perioperative care. Interestingly, in obese patients with GC, adequate preoperative exercise could reduce operative risk [78]. Although more nodes could be retrieved in obese patients [79], the lymph node ratio remained unchanged.

Since BMI was associated with various clinicopathological parameters, it would be important to keep it mind during perioperative management. For instance, for an overweight/obese patient, presurgical blood transfusion would be less necessary. Higher BMI would more often point to the proximal stomach which should be focused on, and a more poorly differentiation grade which would justify the necessity of standardized postoperative therapy and the more careful detection of potential occult metastasis. Further, BMI might also be helpful to guide immunotherapy considering its association with neutrophil-lymphocyte ratio. When considering the prognostic value of BMI, it would be important to make the evaluation in a specific subgroup with specific characteristics (*e.g*., female, AEG, and good differentiation); otherwise, the predictive value in the overall patients would be limited.

The advantages of this investigation lie in its prospective design, large cohort size, long follow-up, use of CSS in survival analyses, detailed and thorough stratification analyses, and appropriate, rigorous and thorough methodology, especially the adjustment strategies. The limitations of this study are that it is a single-institution investigation, and that selection bias might exist with some other potential confounding factors like comorbidities not considered. Moreover, there could be other reasonable groupings of BMI. Notably, the postoperative BMI might be better prognosis-indicative. Besides, in Asia BMIs are generally lower than in the Western world. Specific molecular events were not investigated due to not being part of the original plan of this prospective investigation focusing on the clinical aspects of BMI.

Taken together, this large prospective evidence showed that higher BMI increased surgical time and hospital stay of GC and type II/III AEG patients, and that although overall, preoperative BMI had limited prognostic significance in operated patients, under specific conditions (*e.g*., female, AEG, good differentiation, and thrombocytopenia), BMI might indicate postoperative survival.

## MATERIALS AND METHODS

### Patient cohort

Due to the very high prevalence, the number of patients with upper digestive malignancies resected yearly at Department of General Surgery in The First Affiliated Hospital of Anhui Medical University (FAHAMU) exceeds 1500, potentially ranking 1^st^ worldwide. A total of 700 non-metastasized GC (n=381) and Siewert type-II/III AEG [3] (n=319) patients undergoing radical D2-gastrectomy between January 2009 and December 2010 in Department of General Surgery of FAHAMA were consecutively recruited. Patients ≥15 years, with pTNM stage I-III and pathologically/cytologically-confirmed tumors (imaging-diagnostically confirmed for AEG), with relatively good hepatic and renal functions (serum alanine aminotransferase and aspartate aminotransferase <2.5 times of the upper limit of normal level[ULN], serum total bilirubin <1.5 times of the ULN, serum creatinine ≤1.5 times of the ULN, and international normalized ratio and activated partial thromboplastin time <1.5 times of the ULN) and ECOG scores of 0-2, without severe dysfunctions of important organs (*e.g*., serious uncontrolled cardiopulmonary and neurological dysfunction and hypertension, and active hepatitis B/C virus infection), endocrinal disorders (*e.g*., Cushing's Syndrome and diabetes) or systemic unfits (*e.g*., cachexia, immunodeficiency diseases, and severe psychological disorders), undergoing R0-resectional surgery, and receiving ≥4 cycles of first-line capecitabine-/5-FU-based combination chemotherapy met the inclusion criteria for this prospective cohort. Exclusion criteria were: lymphomas, GIST, sarcomas, type-I AEG, previous cytotoxic/interventional therapies, major abdominal surgery and systemic therapeutics influencing BMI (*e.g*., glucocorticoid and insulin supplements), severe comorbidities, perioperative mortalities due to severe complications, missing records, and rejection of participation by patients. There were 689 eligible patients, and finally 671 with complete follow-up data were analyzed (Table 1). No patients reported receipt of preoperative peripheral blood stimulating regimens or blood product transfusion within 1 month before surgery. This study was approved by the Ethics Committee of FAHAMU, and carried out according to the Helsinki Declaration [80] and Good Clinical Practice [81] guidelines. Written informed consent was obtained from each participant.

Neoadjuvant treatment was not routinely administered in our department, and upfront R0-resection was conducted either openly or laparoscopically for non-metastatic patients. Intraoperative frozen section was routinely performed to ensure resection margins free of malignant residuals. All D2-resections were standard and performed by our experienced group members yearly conducting ≥50 gastrectomies and with surgical practice of ≥5 years. In our department, total gastrectomy was preferred over proximal gastrectomy for AEGs, due to the favorable perioperative outcomes and non-inferior survival [3], and D2 lymphadenectomy was routinely conducted. Roux-en-Y was the commonest anastomosis procedure. Cholecystectomy/splenectomy was performed in case of positive findings (*e.g*., cholecystitis and local invasion) during surgery. Afer R0 resection, all patients received 4-6 cycles of first-line adjuvant combination chemotherapy with oxaliplatin plus 5-FU/leucovorin (FOLFOX) or a prodrug of 5-FU (capecitabine; CapeOX). Radiotherapy was not routinely recommended.

### Clinicopathological parameters

Each patient's body weight and height were measured and recorded upon hospitalization, and BMI was calculated as body weight/height^2^ (unit, kg/m^2^). Based on preoperative conditions, the participants were categorized into underweight (BMI<18.5), normal-weight (BMI=18.5-22.9), overweight (BMI=23-24.9), and obese (BMI≥25) groups according to the Asian standards [82, 83]. Preoperatively, gastroscopy, barium meal, CT and/or MRI assessments were routinely performed, forming the basis of tumor location and clincal staging. Tumor length, pathological type, Borrmann type for advanced diseases, differentiation, harvested and metastatic lymph nodes, neuro-invasion, and tumor thrombosis were obtained from the pathological report, and tumor pTNM stage was according to the TNM classification system (7^th^ version) by AJCC/UICC [84] with recoding done when necessary. Surgical parameters (*e.g*., excision and reconstruction methods, and duration) were based on the surgery and anesthesia records. All patients’ peripheral blood samples were collected into tubes 2-3 days pre-operation, and all blood measurements were conducted within 0.5 hour after blood collection. Pretreatment peripheral blood parameters were obtained from the clinical laboratory test results. Anemia was defined as hemoglobin <130 g/L in men and <120 g/L in women according to WHO, and thrombocytopenia as <140×10^9^/L. Neutrophil-lymphocyte ratio (NLR), which is potentially prognostically significant [55], was calculated as the ratios of the absolute counts of neutrophil to lymphocyte.

### Follow-up

All participants were prospectively followed-up until December 2016, which was conducted in regular intervals according to our standard protocols (every 3 months for the initial 2 postoperative years, every 6 months during years 3-5, and every year thereafter). Patients’ assessments routinely comprised clinical assessments, laboratory examinations, and imaging evaluations. Patients’ relatives were encouraged to report any endpoint events immediately through telephone contact.

### Statistical analysis

Descriptive statistics were used for the overall patients and the four BMI groups, and comparisons of demographical and clinical parameters among the groups were performed using *χ^2^* test for measurement data and Analysis of Variance test for count data. The multivariable logistic regression model was applied to investigate BMI-associated factors, adjusting for gender, age, preoperative hemoglobin, platelet and NLR, tumor location, pathology, differentiation, length, pT stage, metastatic-harvested lymph node ratio, neuro-invasion, and tumor thrombosis.

Cancer-specific survival (CSS) was the primary endpoint, and was defined as the interval between resection and GC-/AEG-associated mortality/last follow-up. The CSS-associated clinicopathological parameters were explored first using univariate Cox analysis applying continuous data, and further by the multivariable Cox regression models adjusting for gender, age, BMI, presurgical hemoglobin, platelet and NLR, surgery type (open/laparoscopic), resection and reconstruction types, cholecystectomy, splenectomy, tumor location, pathology, differentiation, length, pT stage, positive-harvested lymph node ratio, neuro-invasion, and thrombosis. The multivariable variable Cox regressions were further used to assess associations of underweight, overweight, and obesity versus normal-weight with CSS in various subgroups according to gender, age group, tumor location, differentiation, pTNM stage, and preoperational anemia and thrombocytopenia. R (version 3.3.2, Vienna, Austria) was used for data analyses, with two-sided *P*<0.05 indicating statistical significance, and *P*<0.01 strong significance.
